# Effects of Cybervictimization on the Mental Health of Primary School Students

**DOI:** 10.3389/fpubh.2021.588209

**Published:** 2021-05-24

**Authors:** Francesc Sidera, Elisabet Serrat, Carles Rostan

**Affiliations:** Department of Psychology, University of Girona, Girona, Spain

**Keywords:** bullying, cyberbullying, mental health, primary school, strengths and difficulties, behavior

## Abstract

Although many studies have addressed the consequences of cyberbullying on mental health in secondary school, there is a lack of research in primary education. Moreover, most students who are cybervictims also suffer from traditional bullying, and studies on cyberbullying do not always control for the effects of the latter. The aim of our study is therefore to address the possible effects of cyberbullying on different aspects of the life and behavior of students in Years 3 to 6 of primary school. The sample consisted of 636 students attending 38 schools, as well as their parents. Children responded to a bullying and a cyberbullying questionnaire (the EBIPQ and ECIPQ, respectively), and their parents responded to three questionnaires: the Strengths and Difficulties Questionnaire (SDQ), a sociodemographic questionnaire, and one on children's experiences related to bullying and cyberbullying. The results reveal that 14.4% of the children, mostly boys, had suffered at least one online aggression in the previous 2 months. Most of them were also victims of traditional bullying. In this latter group, no differences were found between the SDQ scores reported by cybervictims and those reported by non-cybervictims. In contrast, those cybervictims who were not victims of traditional bullying displayed more difficulties in relation to Conduct problems, Externalizing problems, Home-life impact, and Total difficulties on the SDQ scales. Our results show that cyberbullying affects children's lives as early as primary school, and especially boys, even in children who do not suffer from traditional bullying.

## Introduction

Cyberbullying is defined as a type of bullying that is performed via electronic forms of contact or communication (1). It includes aggressive behaviors of different types, such as those involving written-verbal messages, visual behaviors, exclusion, and impersonation (2). As in the case of traditional bullying, cyberbullying is a hostile behavior that is performed intentionally, repeatedly and within a framework of unequal power between perpetrator and victim (1). Some of the differential characteristics between bullying and cyberbullying that may increase the negative effects of the latter include the fact that in cyberbullying the attacks may come from anywhere, at any time (3), the difficulty of escaping from it, the breadth of the potential audience and the potential anonymity of the bully (4). Moreover, material published online may be shared many times in different places, which potentially increases the harm done to the victim (5).

The aim of the present research is to study the impact of cybervictimization on the mental health of primary school students, as this phenomenon has mostly been studied with adolescents (6). Specifically, our aim is to explore the life impact and behavioral difficulties among primary school children who suffer cyberbullying by considering whether they are also victims of traditional bullying. To this end, we will first review several studies on the prevalence of cyberbullying and its consequences.

Prevalence studies on cybervictimization have mostly been carried out on adolescents. Results vary depending on the study, area and instruments used (7). In the review by Selkie et al. (7), prevalence varied from 3 to 72% in the USA, while a European cross-national study by Tsitsika et al. (8) found a mean prevalence of 21.4%, which was even higher among older teenagers. It is worth highlighting the study conducted by Smahel et al. (9) on children aged between 9 and 16 due to its relevance and size. In this study, pure victimization varied from 1 to 13% depending on the European country, with an average monthly frequency of 5% and sporadic frequency of 9%. However, the study did not report these data specifically for younger children. Overall, the studies by Smahel et al. (9) and Olweus (1) found that despite the differences among countries, the prevalence of cybervictims is lower than 10% in most European countries, and the average is close to 5% in both the USA and EU.

A few studies on cyberbullying have reported data on children under the age of 11. One of these was the research conducted by Livingstone et al. (10), who found a victimization prevalence of 9% in children between the ages of 9 and 16, and specifically of 4% in the age range of 9 to 10 years old. Furthermore, DePaolis and Williford (11), who studied cyberbullying among children in 3rd to 5th grades of elementary school (mean age = 9.4 years), found that 17.7% of the children surveyed had been cyberbullied since the beginning of the school year.

Research reveals a large overlap between traditional bullying and cyberbullying (12), such that victimization only in cyberbullying is rare. For example, in the study by Wolke et al. (13), pure cybervictims made up only 4% (of all those victimized), and the majority of cybervictimizations occurred together with traditional bullying 82.5% of the time. Similarly, in the study by Waasdorp and Bradshaw (14), of all the surveyed teenagers who reported having been bullied, only 4.6% did not report having suffered any form of traditional bullying. However, despite most cybervictims also being victims of traditional bullying, most of the latter do not suffer cyberbullying, so cyberbullying creates few new victims (13). On the other hand, some studies point to a possible higher prevalence of cyberbullying among girls than boys (7, 8, 13, 15).

A review by Kwan et al. (16) shows a strong negative association between cyberbullying and mental health in children and young people. Moreover, Smahel et al. (9) consider frequency to be important when distinguishing between aggressions and bullying, while pointing out that in cyberbullying sporadic incidents may have a significant impact on the well-being of the victim, as they may reach a large audience and can easily remain on the Internet. Hence, despite traditional bullying being more frequent, the differential characteristics between bullying and cyberbullying may also have different consequences. As already explained, we intend to study these differential consequences in greater depth for an understudied age range.

According to some authors, what matters most when it comes to the consequences of bullying or cyberbullying is whether the person has suffered multiple forms of them (13, 17). By way of example, Wolke et al. (13) found that the impact of cyberbullying on mental health is similar to that of traditional bullying (similar to the effects of both direct and relational bullying), while also finding that adolescents who are victims of different forms (who suffered direct and relational bullying simultaneously, for example, or those who suffered them both in addition to cyberbullying) have lower self-esteem and more behavioral problems than those who suffered just one form of victimization. Similarly, Kowalski and Limber (18) found similar negative consequences of traditional bullying and cyberbullying for the physical, psychological, and academic domains.

With regard to the negative effects of cyberbullying, the study by Waasdorp and Bradshaw (14) found that cyberbullied adolescents had a higher risk of suffering multiple forms of bullying, especially relational forms, and of having more externalizing (aggressiveness/irritability...) and internalizing (depression, anxiety...) symptoms. They also found that older teenagers have a higher probability of suffering both bullying and cyberbullying. On the other hand, Smahel et al. (9) found that 44% of children and adolescents who were victims of cyberbullying reported feeling very upset or fairly upset when asked about the last time they were treated in a hurtful or nasty way online. Also, girls reported more harm than boys in this respect.

While several studies on students from 5th to 12th grade have shown that victims of cyberbullying might have consequences such as lower self-esteem, depression, social anxiety or academic problems, studies on primary school children are rare (11). One such study with primary school children was conducted by García-Fernández et al. (19), who found that being a victim of cyberbullying was related to having a negative self-esteem. Nevertheless, they did not report whether those victims were also victims of traditional bullying, which may have influenced their results.

In summary, although the prevalence of cyberbullying may be lower in primary school than in secondary school, it is still necessary to understand the effects it may have.

## Method

### Participants

A total of 636 students enrolled in Years 3 to 6 at 38 primary schools, as well as their parents, participated in the study (52.7% girls). The mean age of the students was 10.09 years (*SD* = 1.18), the age ranging from 8.00 to 12.92 years. A total of 157 students were in Year 3, 144 in Year 4, 184 in Year 5, and 151 in Year 6. This sample of participants was extracted from an initial sample of 4,646 children who responded to the questionnaires, including only those children whose parents responded to the parental questionnaire.

The adults who participated in the study comprised the mother of the child in 64.9% of cases, the father in 18% of cases, both the mother and father in 15.3% of cases, and individuals other than the mother or father in 1.8% of cases (e.g., foster mother, mother's partner, etc.).

### Instruments

The children responded to a bullying and a cyberbullying questionnaire, while the parents responded to a parental questionnaire. These are explained below:

(a) Bullying questionnaire. The European Bullying Intervention Project Questionnaire (EBIPQ) (20) is an instrument that evaluates traditional bullying through seven victimization items and seven aggression items, although only the victimization items were considered in the present study. Children are asked which situations they have experienced over the past 2 months and respond on a Likert scale with five options (0 = No; 1 = Yes, once or twice; 2 = Yes, once or twice a month; 3 = Yes, around once a week; and 4 = Yes, more than once a week). In our study, Chronbach's alpha for the victimization items equaled 0.824.

(b) Cyberbullying questionnaire. A reduced version of the European Cyberbullying Intervention Project Questionnaire (ECIPQ) (20) was used. While the original instrument contains 22 items, the version used here has 12 items (six on cybervictimization and six on cyberbullying) in order to adapt them better to primary school students. Thus, while some items were retained, some others were erased or combined. Only the cybervictimization items have been used in the present study. Chronbach's alpha for the 6 cybervictimization items was 0.774.

c) Parental questionnaires. Parents responded to three questionnaires. In the first, they were asked about their children's experiences in relation to bullying and cyberbullying situations. The questions were adapted from the interview guide that Sawyer et al. (21) used to interview parents of children who had been victimized. In the second questionnaire, parents were asked sociodemographic questions. The third questionnaire was the Strengths and Difficulties Questionnaire [SDQ; (22)]; specifically, we used the Spanish and Catalan double-sided version with impact supplement for the parents of 4–17 year olds. This questionnaire is a brief behavioral screening questionnaire consisting of 25 items, divided into five scales with five items each: emotional symptoms, conduct problems, hyperactivity/inattention, peer relation problems and prosocial behavior. Chronbach's alpha for these 25 items was 0.761. Apart from these items, the impact supplement asks parents whether they believe their child has difficulties, since when, whether these difficulties cause distress to the child, and whether they affect the child in the following areas: home-life, friendships, school learning, and leisure activities. Chronbach's alpha for these 5 items of the impact scale was 0.716.

### Procedure

A representative sample of students enrolled in Years 3 to 6 at state-run and private schools in Catalonia (Spain) was selected. The parents of the children were also asked to respond to a questionnaire, and in the present study we only included those children whose parents responded to the parents' questionnaire. Families were informed of the objectives of the study, and they provided written informed consent. Both children and parents were given the opportunity to respond to the questionnaires in either Catalan or Spanish.

The children responded to the questionnaires in their own classrooms, either on paper or in an online version (depending on the teacher's decision); the majority of classes used the paper version. In these cases, when children finished responding to the questionnaire, they were asked to put it inside an envelope and seal it. In most schools, the project researchers were present when administering the questionnaires to the children, although five of the schools chose to administer them on their own.

Regarding the parents, they were sent a link through which they could access the questionnaires online. They were each asked to enter a personal code for their questionnaire, thus linking it to the child's questionnaire while maintaining anonymity.

In the present study, those students who reported having been subjected to at least one cybervictimization behavior of any frequency on the cyberbullying questionnaire were labeled as “cybervictims.” On the other hand, those who reported having been the subject of at least one victimization behavior with a minimum frequency of “once or twice per month” were considered “victims of traditional bullying.”

In relation to the SDQ scores, the possible range of scores for the five symptoms scales (emotional problems, conduct problems, hyperactivity/inattention, peer problems, and prosocial scale) was 0–10. The Externalizing and Internalizing scores ranged from 0 to 20. The Externalizing score included the conduct and hyperactivity/inattention scales, and the Internalizing score the emotional and peer problem scales. The Total difficulties score included all scales for the Internalizing and Externalizing scores, and ranged from 0 to 40. In addition, the range of the SDQ impact scores was 0–2 and the Impact total score 0–10.

The approval of the institutional review board (IRB) from the University of Girona was obtained for conducting the study (code: CEBRU0016-2018)

In Table 1, the column “Group comparison” shows which differences were significant at level “*p* < 0.05” after carrying out ANOVAs (we compared only the following groups: a and b, c and d, and b and d). Contrast statistics are provided in the text.

**Table 1:** Comparison of means (and SD) between cybervictims and non-cybervictims among victims and non-victims of traditional bullying.

	**Not victims of traditional bullying**		**Victims of traditional bullying**		**ANOVA group comparison**
	**(a) Not cybervictims**	**(b) Cybervictims**	**(c) Not cybervictims**	**(d) Cybervictims**	
**SDQ symptoms scores**					
Emotional problems scale	1.91 (1.93)	2.33 (1.88)	2.64 (2.30)	3.04 (2.67)	
Conduct problems scale	1.19 (1.32)	1.70 (1.33)	1.62 (1.60)	2.04 (1.50)	a < b
Hyperactivity scale	2.84 (2.34)	3.46 (2.38)	3.69 (2.46)	3.80 (2.43)	
Peer problems scale	1.29 (1.61)	1.51 (1.74)	1.89 (1.93)	1.93 (1.89)	
Prosocial scale	8.88 (1.37)	8.64 (1.53)	8.54 (1.47)	8.41 (1.89)	
Externalizing score	4.02 (3.20)	5.27 (3.18)	5.30 (3.65)	5.85 (3.23)	a < b
Internalizing score	3.21 (3.01)	3.73 (3.07)	4.52 (3.66)	4.98 (3.83)	
Total difficulties score	7.22 (5.24)	9.08 (5.60)	9.83 (6.29)	10.83 (5.67)	a < b
**SDQ impact scores**					
Upset-Distress	0.23 (0.49)	0.05 (0.23)	0.38 (0.62)	0.53 (0.82)	b < d
Home-Life	0.08 (0.31)	0.32 (0.67)	0.14 (0.40)	0.33 (0.58)	a < b
Friendships	0.13 (0.40)	0.26 (0.56)	0.25 (0.55)	0.40 (0.68)	
Classroom learning	0.25 (0.55)	0.53 (0.77)	0.41 (0.63)	0.53 (0.68)	
Leisure activities	0.09 (0.37)	0.16 (0.37)	0.16 (0.46)	0.24 (0.54)	
Impact Total	0.81 (1.42)	1.31 (1.97)	1.28 (1.77)	1.95 (2.30)	

## Results

Of the 636 children in the sample, a total of 90 were considered as cybervictims (14.4%). Of these, only 33.3% were girls. The Chi-Square showed that the variables cybervictim and gender were significantly related (χ^2^ = 15.871; *p* < 0.001). Furthermore, of the 90 cybervictims, 48 (53.3%) were also considered as victims of traditional bullying. Among those children who were not cybervictims, a total of 143 were considered as victims of traditional bullying and 403 were not. The Chi-Square test showed that the variables cybervictim and victim of traditional bullying were significantly related (χ^2^ = 27.090; *p* < 0.001). Thus, while the percentage of cybervictims among victims of traditional bullying was 25.13%, the percentage of cybervictims among non-victims of traditional bullying was of only 9.44%.

In order to study the effects of cyberbullying on children, an ANOVA was performed to compare those considered cyberbullied and those considered non-cyberbullied in both the group of children who were victims of traditional bullying and the group of children who were not such victims (see Table 1 for descriptive scores). In the latter group, the ANOVA showed differences between cybervictims and non-cybervictims (comparison between groups *a* and *b*) on the conduct problems scale [*F*_(1, 423)_ = 5.133; *p* = 0.024; η^2^ = 0.12], in the Externalizing score [*F*_(1, 419)_ = 5.106; *p* = 0.024; η^2^ = 0.12], in the Total difficulties score [*F*_(1, 413)_ = 54.095; *p* = 0.044; η^2^ = 0.10], in the home-life impact [*F*_(1, 143)_ = 6.119; *p* = 0.015; η^2^ = 0.04], and close to significant differences in classroom learning [*F*_(1, 143)_ = 3.603; *p* = 0.060; η^2^ = 0.02]. In contrast, no differences were found between cybervictims and non-cybervictims in the group of children who were victims of traditional bullying (comparison between groups *c* and *d*) on the SDQ scores (*p* > 0.05 in all cases).

We also performed an ANOVA to compare the two groups of cybervictims (those who had been subjected to traditional bullying and those who had not). In this case, the scores of the two groups were very similar, except in the upset-distress impact score, where higher scores were reported for the group of cybervictims with traditional bullying than those reported for cybervictims without such bullying [*F*_(1, 38)_ = 5.932; *p* = 0.020; η^2^ = 0.13].

## Discussion

Our first observation is that cybervictimization occurred in children in Years 3 to 6 of primary school, similar to the finding by DePaolis and Williford (11). Also, it was more prevalent among boys than girls, another finding matching that of DePaolis and Williford (11) at similar ages. Studies with older samples have usually found a higher prevalence among girls than boys [see: Selkie et al. (7), Tsitsika et al. (8), Wolke et al. (13), Smith et al. (15), UNESCO (23)]. Therefore, it is possible to deduce that while the prevalence of cybervictims is higher among girls in secondary school, in primary school it may be higher in boys. If this is confirmed by future studies, the reasons why primary school boys suffer more cyberbullying than girls should also be investigated. With regard to this, DePaolis and Williford (11) found that boys were more likely to be cybervictimized through online games than girls.

Furthermore, similarly to previous studies with adolescents (12, 13), in our study with primary school children we found that the likelihood of being a cybervictim was higher among those children who suffer from traditional bullying compared to those who do not. For victims of traditional bullying, the added problem of cyberbullying did not imply any additional difficulties. Although some other studies have not found a negative additive effect of offline and online victimization, most studies on the subject have (24). For example, the research that Vieno et al. conducted on more than 24,000 adolescents found that cybervictimization experiences increased the likelihood of suffering psychological and somatic symptoms, even when traditional bullying was taken into account (25). Furthermore, the effects of cyberbullying were found to be higher for *frequent* victims than for *occasional* ones. In a similar vein, a meta-analysis by Gini et al. (26) found that cyberbullying made a unique contribution to the internalizing problems suffered by adolescents. Although it is possible that the unique effects of cybervictimization are difficult to detect without a very large sample (26), the difference between these studies and ours might also be due to developmental reasons. We must also consider that these consequences are usually measured using self-report instruments in adolescents, whereas the age of our young sample led us to use parental reports. It is also worth noting that different studies have used very different instruments, so more research is needed to better understand the unique effects of cyberbullying in primary school children. In addition to the above, the upset-distress score among cybervictims in our study was significantly higher in children who also suffered from traditional bullying. This leads to the question, why might being a traditional victim worsen the situations of cybervictims but not vice versa? One explanation would be that we used a less strict criterion for defining cybervictims. However, a simple act of cyberbullying may reach many people or have a permanent impact over time (27), so cybervictimization and traditional victimization are not easily comparable in terms of intensity or frequency.

On the other hand, in the group of children who were not victims of traditional bullying, being a cybervictim did have an effect on some behavioral and impact scores on the SDQ. Among this group of children, being a cybervictim implied higher scores in conduct problems and Externalizing problems (which, in addition to conduct problems, include hyperactivity/inattention problems). Prior studies had reported significant positive correlations between cybervictimization and externalizing symptoms as measured by the SDQ (28). Our study has confirmed this relationship in primary school children who do not suffer traditional bullying, although we cannot say whether these problems already occurred before suffering cyberbullying. In any case, being solely a cybervictim had an impact on the home life of the child. These results show that cyberbullying might already be affecting children's lives in primary school, even among those who do not suffer from traditional bullying. Therefore, the issue of cyberbullying should be addressed with great rigor (29).

One limitation of our study is that some parents did not respond to the questionnaire, so the characteristics of our sample might differ from the potential initial sample of parents. Also, the relationship between the symptoms scores on the SDQ and victimization should be interpreted with caution, since there could be a two-way influence. Despite these limitations, our study has some strong points. Firstly, it is one of the few to analyse the unique consequences of cyberbullying in primary school students. Secondly, we studied its effects on children who both suffer and do not suffer from traditional bullying. In this respect, our results revealed no differences when comparing cybervictims and non-cybervictims among those children who were victims of traditional bullying. Hence, research aimed at studying the effects of cyberbullying should control for traditional bullying, otherwise, the overlap between the two could lead to the effects of traditional bullying being interpreted as effects of cyberbullying.

To sum up, then, we found that, contrary to what happens in secondary school, cyberbullying in primary school is more prevalent among boys than girls. Moreover, the impact of cyberbullying was found to be higher in children who did not suffer from traditional bullying than in those who did. Also, according to Kwan et al. (16), future research should carry out longitudinal studies in this field in order allow us to understand the long-term consequences of cyberbullying in primary school, as well as its causal relationship with children's mental health and psychosocial well-being.

## Data Availability Statement

The raw data supporting the conclusions of this article will be made available by the authors, without undue reservation.

## Ethics Statement

The studies involving human participants were reviewed and approved by Comitè d'Ètica i Bioseguretat de la Recerca de la Universitat de Girona. Written informed consent to participate in this study was provided by the participants' legal guardian/next of kin.

## Author Contributions

All authors have contributed a similar amount in all sections of the manuscript.

## Conflict of Interest

The authors declare that the research was conducted in the absence of any commercial or financial relationships that could be construed as a potential conflict of interest.

## References

[1] Olweus D. School bullying: development and some important challenges. Annu Rev Clin Psychol. (2013) 9:751–80. 10.1146/annurev-clinpsy-050212-185516 23297789

[2] Nocentini A Calmaestra J Schultze-Krumbholz A Scheithauer H Ortega R Menesini E. Cyberbullying: labels, behaviours and definition in three European countries. J Psychol Couns Sch. (2010) 20:129–42. 10.1375/ajgc.20.2.129

[3] Tokunaga R. Following you home from school: a critical review and synthesis of research on cyberbullying victimization. Comput Human Behav. (2010) 26:277–87. 10.1016/j.chb.2009.11.014

[4] Slonje R Smith PK. Cyberbullying: another main type of bullying? Scand J Psychol. (2008) 49:147–54. 10.1111/j.1467-9450.2007.00611.x 18352984

[5] Kowalski RM Giumetti GW Schroeder AN Lattanner MR. Bullying in the digital age: a critical review and meta-analysis of cyberbullying research among youth. Psychol Bull. (2014) 140:1073–37. 10.1037/a0035618 24512111

[6] Williford A DePaolis KJ. Validation of a cyber bullying and victimization measure among elementary school-aged children. Child Adolesc Social Work J. (2019) 36:557–70. 10.1007/s10560-018-0583-z

[7] Selkie EM Fales JL Moreno MA. Cyberbullying prevalence among US middle and high school–aged adolescents: a systematic review and quality assessment. J Adolesc Health. (2016) 58:125–33. 10.1016/j.jadohealth.2015.09.026.17 26576821 PMC4724486

[8] Tsitsika A Janikian M Wójcik S Makaruk K Tzavela E Tzavara C. Richardson C. Cyberbullying victimization prevalence and associations with internalizing and externalizing problems among adolescents in six European countries. Comput Human Behav. (2015) 5:1–7. 10.1016/j.chb.2015.04.048

[9] Smahel D Machackova H Mascheroni G Dedkova L Staksrud E Ólafsson K Livingstone S. EU Kids Online 2020: Survey Results From 19 Countries. London: EU Kids Online (2020). 10.21953/lse.47fdeqj01ofo

[10] Livingstone S Haddon L Görzig A Ólafsson K. Risks and Safety on the Internet: The Perspective of European Children: Full Findings and Policy Implications From the EU Kids Online survey of 9-16 Year Olds and Their Parents in 25 Countries. EU Kids Online. London: EU Kids Online Network (2011).

[11] DePaolis K Williford A. The nature and prevalence of cyber victimization among elementary school children. Child Youth Care Forum. (2015) 44:377–393. 10.1007/s10566-014-9292-8

[12] Del Rey R Elipe P Ortega-Ruiz R. Bullying and cyberbullying: overlapping and predictive value of the co-occurrence. Psicothema. (2012) 24:608–13. Available online at: http://www.psicothema.com/psicothema.asp?id=4061 23079359

[13] Wolke D Lee K Guy A. Cyberbullying: a storm in a teacup? Eur Child Adolesc Psychiatry. (2017) 26:899–908. 10.1007/s00787-017-0954-6 28188382 PMC5532410

[14] Waasdorp TE Bradshaw CP. The overlap between cyberbullying and traditional bullying. J Adolesc Health. (2015) 56:483–8. 10.1016/j.jadohealth.2014.12.002 25631040

[15] Smith PK Mahdavi J Carvalho M Fisher S Russell S Tippett N. Cyberbullying: its nature and impact in secondary school pupils. J Child Psychol Psychiatry. (2008) 49:376–85. 10.1111/j.1469-7610.2007.01846.x 18363945

[16] Kwan I Dickson K Richardson M MacDowall W Burchett H Stansfield C. Cyberbullying and children and young people's mental health: a systematic map of systematic reviews. Cyberpsychol Behav Soc Netw. (2020) 23:72–82. 10.1089/cyber.2019.0370 31977251 PMC7044782

[17] Cross D Lester L Barnes A. A longitudinal study of the social and emotional predictors and consequences of cyber and traditional bullying victimisation. Int J Public Health. (2015) 60:207–17. 10.1007/s00038-015-0655-1 25645100

[18] Kowalski RM Limber SP. Psychological, physical, and academic correlates of cyberbullying and traditional bullying. J Adolesc Health. (2013) 53:S13–20. 10.1016/j.jadohealth.2012.09.018 23790195

[19] García-Fernández CM Romera-Félix EM Ortega-Ruiz R. Cyberbullying en educación primaria: factores explicativos relacionados con los distintos roles de implicación. Pyschol Soc Educ. (2017) 9:251–62. 10.25115/psye.v9i2.697

[20] Ortega-Ruiz R Del Rey R Casas JA. Evaluar el bullying y el ciberbullying validación española del EBIP-Q y del ECIP-Q. Psicol Educ. (2016) 22:71–9. 10.1016/j.pse.2016.01.004

[21] Sawyer JL Mishna F Pepler D Wiener J. The missing voice: parents' perspectives of bullying. Child Youth Serv Rev. (2011) 33:1795–803. 10.1016/j.childyouth.2011.05.010

[22] Goodman R. The strengths and difficulties questionnaire: a research note. J Child Psychol Psychiatry. (1997) 38:581–6. 10.1111/j.1469-7610.1997.tb01545.x 9255702

[23] UNESCO. Behind the Numbers: Ending School Violence and Bullying. France: UNESCO (2019).

[24] Glüer M Lohaus A. Frequency of victimization experiences and well-being among online, offline, and combined victims on social online network sites of German children and adolescents. Front Public Health. (2015) 3:274. 10.3389/fpubh.2015.00274 26734598 PMC4683168

[25] Vieno A Gini G Lenzi M Pozzoli T Canale N Santinello M. Cybervictimization and somatic and psychological symptoms among Italian middle school students. Eur J Public Health. (2015) 25:433–7. 10.1093/eurpub/cku191 25465914

[26] Gini G Card NA Pozzoli T. A meta-analysis of the differential relations of traditional and cyber-victimization with internalizing problems. Aggress Behav. (2018) 44:185–98. 10.1002/ab.21742 29160943

[27] Pozzoli T Gini G. Behavior during cyberbullying episodes: initial validation of a new self-report scale. Scand J Psychol. (2020) 61:22–9. 10.1111/sjop.12517 30690730

[28] Longobardi C Gullotta G Ferrigno S Jungert T Thornberg R Settanni M. Cyberbullying and cybervictimization among preadolescents: does time perspective matter? Scand J Psychol. (2020) 62:259–66. 10.1111/sjop.12686 33048359

[29] Bonanno RA Hymel S. Cyber bullying and internalizing difficulties: above and beyond the impact of traditional forms of bullying. J Youth Adolesc. (2013) 42:685–97. 10.1007/s10964-013-9937-1 23512485

